# Sex difference and immunosenescence affect transplantation outcomes

**DOI:** 10.3389/frtra.2023.1235740

**Published:** 2023-08-22

**Authors:** Ryoichi Maenosono

**Affiliations:** Department of Urology, Faculty of Medicine, Osaka Medical and Pharmaceutical University, Takatsuki, Japan

**Keywords:** aging, senescence, sex difference, kidney transplantation, end-stage renal disease

## Abstract

Kidney transplantation is a well-established alternative to renal replacement therapy. Although the number of patients with end-stage renal disease (ESRD) is increasing, the availability of kidney for transplantation is still insufficient to meet the needs. As age increases, the prevalence of ESRD increases; thus, the population of aged donors and recipients occupies large proportion. Accumulated senescent cells secrete pro-inflammatory factors and induce senescence. Additionally, it is gradually becoming clear that biological sex differences can influence aging and cause differences in senescence. Here, we review whether age-related sex differences affect organ transplant outcomes and what should be done in the future.

## 1. Introduction

It is well known that the number of patients with end-stage renal disease (ESRD) requiring renal replacement therapy is increasing globally (1, 2), while the number of patients on kidney transplant waiting list is also increasing. The main barrier to kidney transplantation is severe shortage of donors. To increase the pool of organ donation, marginal kidneys from extended-criteria donors (ECDs) have been used in the past two decades. An ECD is often defined as a deceased donor >60 years old or 50–59 years old meeting at least two of the following three criteria: cerebrovascular cause of death, terminal serum creatinine >1.5 mg/dl, or history of hypertension (3). Recently, the number of aging individuals has been increasing worldwide, especially in developed countries, and this increase will continue for the time being. In 1950, no country had >11% of its population aged ≥65 years; in contrast, the highest population with older individuals was 18% in 2000. Moreover, by 2050, the percentage of older individuals is expected to reach 35%, which is estimated to be higher than that of adolescents aged 10–24 (3). With the aging population worldwide, the prevalence of ESRD is expected to increase; thus, aged donors and recipients will be involved in kidney transplantation. However, organs from older donors are underutilized, frequently discarded, or not considered (4, 5).

Aging can affect not only physical changes but also the immune response to organ transplantation. Accumulated evidence suggests that increased donor age is a significant risk factor for adverse outcomes, including more frequent rejections due to augmented immunogenicity during aging (6, 7), in addition to increased rates of chronic allograft dysfunction in kidney, heart, and lung transplantations (8). It is well known that senescent cells accumulate with aging and drive the immunogenicity of older organs, which is linked to the accumulation of cell-free mitochondrial DNA that accelerates alloimmune responses (9). Depletion of senescent cells ameliorates a wide range of age-associated disabilities and diseases (10). In contrast, an attenuated acquired immune response to donor organs is observed with recipient aging, which is related to the graft outcome. For instance, senescence in T cells upregulates programmed death protein (PD-1), T cell immunoglobulin and mucin domain containing-3, and lymphocyte activation gene-3, with impaired cytotoxicity and cytokine production, thereby orchestrating lower antigen-specific reactions in older individuals (11).

Simultaneously, sex difference in aging may influence the accumulation of senescent cells. Currently, insight into gendered innovations is recognized as an important factor in investigations. In 2016, the National Institutes of Health (NIH) established the Sex as a Biological Variable policy (12). Although the effect of sex differences in transplantation is influenced by various factors and need not describe a single factor, in comparison with males, female sex with aging could delineate T cell immunity and potentially impact graft survival, owing to changing sex hormones in a lifetime.

Here, we introduce potential mechanisms and consequences of senescence that are related to sex and discuss clinically relevant aspects of senescent cell spread when transplanting older organs. These interactions have seldom been discussed. Therefore, new insights may be provided by transplantation studies.

## 2. Influences of biological sex difference in a disease

It is well recognized that biological sex can affect the incidence of diseases; females are more susceptible to autoimmune diseases (13), cardiovascular disorders (14), and neurodegenerative conditions (15). In contrast, male sex has been shown to be a risk factor for infections, including COVID-19 (16, 17), obstructive coronary artery disease (18), and Parkinson's disease (19).

Sex differences are often defined as differences at the chromosomal level and sex-related hormones, leading to differences in physical characteristics and behavior. Chromosomal difference is thought to result in sex difference. The X chromosome harbors several genes related to immunity, including *TLR7*, *FOXP3*, *CD40l*, and *IL2RG*. In females, mosaicism, which causes X chromosome inactivation, is one mechanism that modulates the normal expression of X-related genes and immune responses, while males have only one X chromosome; therefore, mosaicism does not appear in males (20). It is known that as many as 23% of genes on the X chromosome can escape inactivation, by mechanisms involving Xist RNA and PCGF3/5 (Polycomb group RING finger) (21). Therefore, several immunity-related genes may express at higher levels in females (22, 23), causing inflammation and autoimmune disease.

Aging is also recognized as an influencing factor for both sex hormones. After adolescence, the upregulation of testosterone and estradiol levels by gonadotropins physically characterizes each sex. With aging, circulating testosterone levels decline in both men and women (24, 25), leading to osteoporosis in both sexes (26). The correlation between immunological properties and sex hormones has been thoroughly investigated, particularly in relation to autoimmune diseases.

Estrogen, for instance, modulates Th1 cells (27, 28) through the interaction of the estrogen receptor (ER) with the promoter region of the interferon (IFN)-γ gene and through the induction of the transcription factor T-bet (27, 28). ER alpha (Erα) knock out in the T cell, CD4-creER^flu/flu^, limited the accumulation and production of inflammatory cytokines and influenced the fate of the cell compared to a control model, thereby showing ER and estrogen axis strongly connecting acquired T cell immunity (29). Moreover, castrated female mice with low dose of 17-β estradiol replacement showed striking increase in antigen-specific Th1 response compared with an ovariectomized female model, which showed that estrogen administration could module CD4^+^ T cell activity in a dose specific manner (30); however, elevated estrogen levels have been shown to promote an augmented Th2 cell response (31). Immune effects of estrogen appear to be dose-dependent, as highly elevated estrogen levels during pregnancy induce regulatory T cells (Tregs), contributing to an intact pregnancy of a semi-allogeneic fetus (32).

However, there is evidence that the effect of estradiol on immune response is equally capable of the opposite. In particular, menopause and ovariectomy negatively affect osteogenesis, thereby exacerbating bone loss. Bone marrow mesenchymal stem cell co-cultured with ovariectomized T cell, which expresses higher tumor necrosis factor alpha (TNF-α), shoed reduced levels of osteogenic differentiation and reduced expression of Runx2 and osteocalcin compared with sham mice (33). Both opinions have something in common: pregnancy level estradiol may induce anti-inflammatory effects by inhibiting Th1 cell response and increasing Treg number (3).

Therefore, while some studies have reported that decreased estrogen levels could introduce fewer inflammatory effects, whether depleted estrogen favors the establishment of inflammation may ultimately depend on a variety of context-specific factors.

## 3. Effect of sex and aging on organ transplantation

**Figure 1:**
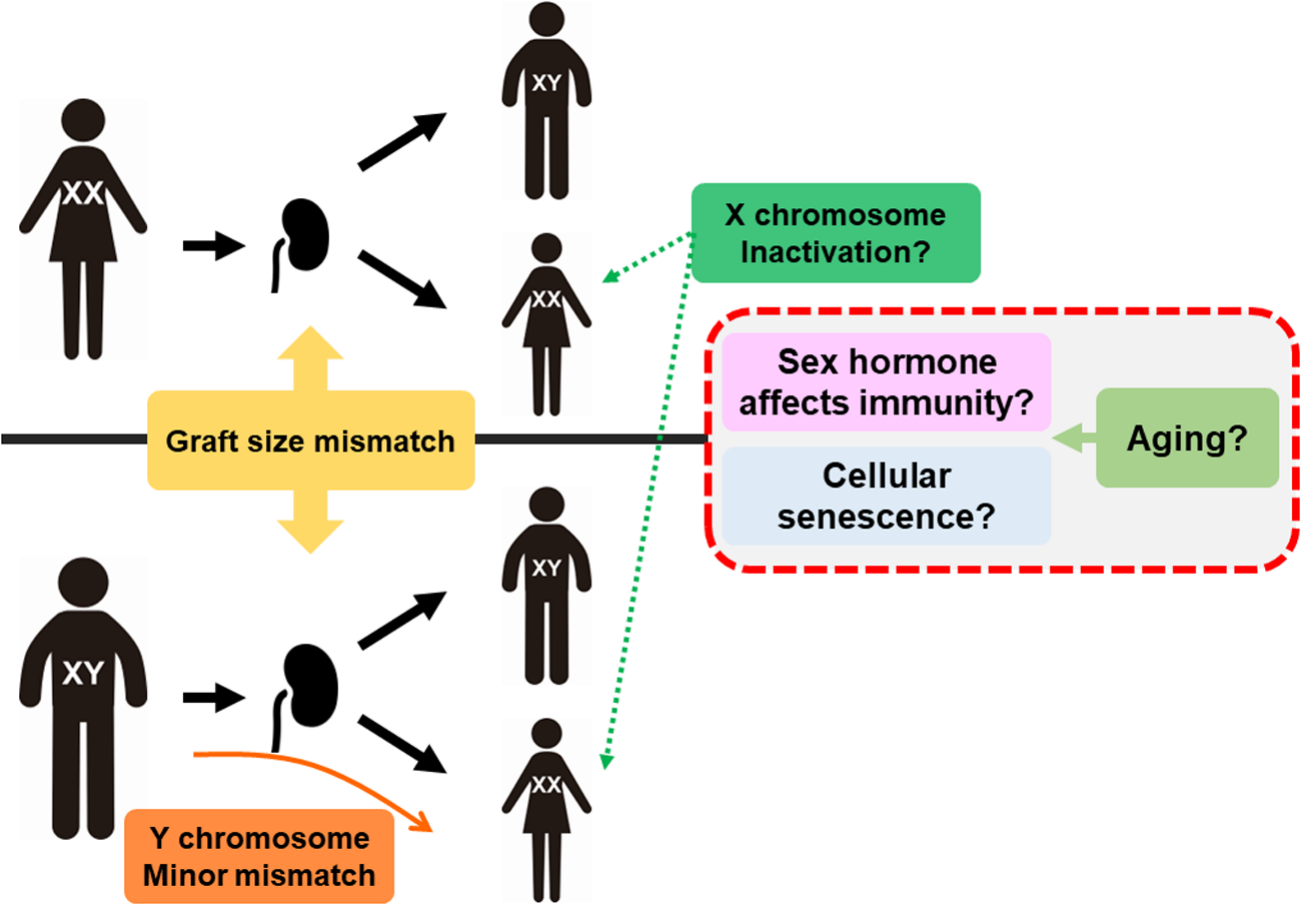
Mechanism of sex and aging affecting transplantation. Graft size mismatch is considered as nephron counts mismatch in males and females. For female recipients, Y chromosome is minor histo-incompatible antigen but also inactivation of X chromosome may lead to higher expression of several genes related to immunity, such as *TLR7*. Aging can affect the sex hormone balance and accumulation of senescence cells, resulting in alloimmune response changes.

With respect to transplantations, it has been controversial whether the relationship between sex difference can cause differences in kidney graft outcomes because those cannot be described by only one hormonal factor (Figure 1). Donor/recipient size mismatch between both sexes is also recognized as having a strong influence because nephron counts have been shown to contribute to inferior graft function (34, 35). Chromosomal mismatch is contributing to the outcome. Female recipients with XX as the sex chromosome have a higher graft loss rate when receiving sex chromosome-incompatible (XY) transplantation (36, 37).

Although several factors related to sex differences are complexly intertwined, sex hormones, especially female hormones, are thought to be dramatically upregulated and downregulated during a lifetime, such as adolescence, pregnancy, and menopause. Although the effects of sex hormones on immunity have been recognized, their impact on alloimmune responses over a lifetime remains unclear. In this direction, recent studies have accumulated supporting evidence about sex difference with aging.

A study of kidney transplantation among 3–29-years-old showed that Th1 (IFNγ^+^IL-4^−^L-17A^−^) and Th17 (IL-17A^+^) frequencies within CD4^+^ T cells could be higher at older ages, but the frequencies of FOXP3^+^ Treg cells in CD4^+^CD25^+^CD127^−^ T cells were lower in females than in males (38). This report did not show serum estrogen levels; however, women in their twenties could be estimated to have higher estrogen levels than pre-adolescent and post-menopausal women. Another study demonstrated that younger female recipients represented by 15–35-years-old, had inferior death-censored graft survival, whereas graft survival was superior in older female recipients (55–75-years-old). Experimental models also showed prolonged graft survival in young ovariectomized females and increased the number of estradiol recipients. Although increasing estradiol concentrations can prompt a switch from naïve CD4^+^ T cells to Th1 cells, high physiological estradiol concentrations dampen Th1 responses and promote Tregs (39).

Although sex hormones are directly associated with aging and have several effects on transplant immunology, the next question is whether cellular and immune senescence in an organ transplantation could be affected by sex difference in an aging-specific manner.

In organ transplantation, recipients of old organs show lower graft survival with an increasing frequency of rejection, especially in young recipients (6). In addition to the increasing number of aging donors, ischemic reperfusion injury, which is inevitable from extraction to transplantation, is associated with accumulated senescent cells in the donor organ. In support of this, cell-free mitochondrial DNA released by senescent cells from donor organs can promote an inflammatory response by stimulating TLR-9, thereby activating dendritic cellular activity and deteriorating graft outcomes (9). Furthermore, inflammasome and IL-1 signaling are activated in senescent cells and IL-1a reproduces a senescent-associated secretory phenotype (SASP), suggesting that the spread of senescent cells from donor organs could not only induce immunogenicity but also accelerate aging in recipients, similar to the results from an experimental study in which senescent cells were observed surrounding a tissue adjacent to papilloma with p16^Ink4a^ and p21^Cip1^ (40). Additionally, a recent study showed that genetically modified Vav-iCre^+/−^; Ercc1^−/flu^ mice having conditional deletion of endonuclease ERCC1, a crucial DNA repair protein in hematopoietic cells, can induce senescence in the immune system. These mice showed not only an impaired immune system but also non-lymphoid organ damages, suggesting that increased immune senescence promotes systemic aging. Interestingly, normal young mice transplanted splenocytes from Vav-iCre^+/−^; Ercc1^−/flu^ mice also induced senescence, whereas transplantation of young immune cells into this model attenuated their senescence. Rapamycin, a senolytic agent, can improve immune function (41). Additionally, combination treatment with dasatinib and quercetin depleted senescent cells in donor organs from old mice, decreasing SASP factors and prolonging transplant survival (9).

Thus, senolytic agents that can clear senescent cells in donor organs may not only exert anti-inflammatory effects but also restrain a potential transfer of senescence, which has a strong therapeutic potential in organ transplantation; however, does immune senescence in sex differences influence organ transplantation outcomes?

## 4. Does sex difference promote inflammation via cellular senescence?

Generally, cellular senescence is thought to occur in response to exogenous and endogenous inducers during the lifetime of cells, such as telomere erosion (42, 43), oxidative stress, radiation, ultraviolet radiation, and chemical agents (44), and then acquire anti-apoptotic pathways and stability with growth arrest (10). The cellular senescence is caused by generating DNA damage and/or response to damage signaling mechanisms. Telomere erosion can directly initiate a DNA damage response, and chemotherapy and radiation are known to cause single- and double-strand breaks (SSBs and DSBs) (45, 46). Such damages are sensed by protein complexes, then ataxia-telangiectasia mutated and ataxia-telangiectasia and Ras3-related (ATR) proteins are recruited to DNA damage sites and facilitate senescence-associated cell cycle arrest via the p53-p21 pathway (47). Furthermore, the upregulation of p16^ink4a^ causes senescence growth arrest (44). As a result, senescent cells are accumulated in many tissues owing to aging and/or damage. Permanently persistent cellular senescence acquires SASP and exhibit pro-inflammatory factors, consisting of cytokines (IL-6, IL-8, and TNF-a) and chemokines (CCL2 and CCL20) (48).

Current evidence shows that cells from females are more susceptible to DNA damages induced by genotoxic stress and both SSB and DSB repair (SSBR and DSBR, respectively) appear to be lower in these cells than that in cells from males (49). A lower capacity for both SSBR and DSBR was observed in female patients, while cells from females tended to have a higher proportion of senescent cells, and cells from males underwent apoptosis or malignant transformation (50). Estrogen, a sex hormone that characterizes the female sex, prevents cellular senescence by protecting against senescence-inducing DNA damage and inhibiting the senescence establishment pathway; estrogen levels are dramatically reduced in women after menopause. Supporting the results from these reports, estrogen treatment was shown to prevent Lipopolysaccharide/IFN-γ action on human M2 macrophages through NF-γ B release in human macrophages (51). Bone marrow mesenchymal stem cells from ovariectomized mice showed upregulation of p53, p21, and SASP, whereas administration of high levels of estradiol decreased the abundance of senescence properties (p53 and p21) related to the JAK/STAT pathway (52).

Furthermore, inhibition of ER can upregulate the senescence marker β-galactosidase (53). Many lines of evidence support that estradiol could protect against senescence. In contrast, although estrogen is known to indirectly suppress expression of CDKN1A, the gene that encodes p21 through ERα-driven inhibition of ATR and subsequent inactivation of p53 (54), some reports showed that estrogen may promote cellular senescence by modulating the activity of pathways that can upregulate CDKN1A as a result of activated ERα gene expression (55) and persistent reactive oxidative stress, thereby causing DNA damages.

Evidence indicates that senescent cells accumulate with aging, and/or stress can spread in transplantation recipients and then accumulate in their bodies. Senescent cells could differ not only by the donor's sex (females tend to be more senescent) but also by the sex of the recipient, and their sex hormones might upregulate or downregulate SASP. To date, it is unclear whether senolytic agents can be of advantage in males or females; therefore, further studies are needed to verify that biological sex influences cellular senescence in human contexts and reconcile contrasting predictions on how senescence might manifest in women specifically.

## 5. Does senescence-associated T cell (SAT) affected by sex difference influence transplantation outcome?

T cells are responsible for acquired immunity, and they play an important role in kidney transplantation. CD4^+^ and CD8^+^ T cells usually present antigens via antigen-presenting cells, such as macrophages or dendritic cells, and acquire specific effector properties in secondary lymphoid tissue. T cells were initially selected from the thymus to obtain tolerance.

However, in lineage cells, aging causes involution and declining function of the thymus, resulting in decreased T cell production. In total, peripheral T cell number was stable and unaffected by aging, with the thymus contributing more at younger ages, and peripheral T cell expansion contributing more in older individuals (56). T cell population shifts from naïve to memory phenotype T cells in peripheral side (57, 58), which is thought to contribute to T cell immunosenescence, leading to an increased population with poor responsiveness to new antigens (59). Recently, SAT cells with defective proliferation and effector function were broadly investigated in the context of cancer therapy. Findings from a study suggests that inducing T cell senescence is a key strategy used by malignant tumors to evade immune surveillance (11).

SAT cells might also be affected by sex hormone levels. CD44^high^PD-1^+^CD4^+^ SAT cells from gonadal visceral adipose tissues increased in number in ovariectomized female mice than in naïve mice; however, estradiol replacement therapy changed the SAT cell population (60). It has been reported that, during transplantation, CD4^+^IFN-γ^+^ T cells from splenocytes derived from ovariectomy and higher-level estradiol conditions (such as pregnancy) showed lower inflammatory properties, causing prolonged graft survival (39). In this study, ovariectomized CD4^+^ T cells might have a less effective function derived from senescence; however, there was no reference to senescent cells. Therefore, further studies are needed to transfer and apply the findings from an animal model to a human study.

## 6. Discussion

Recent accumulated evidence shows that transplanted senescent cells from older donors can negatively affect organ transplant recipients because of the opportunity for higher graft rejection, greater accumulation of senescence, and SASP. On the recipients' side, there is controversy whether T cell senescence can positively or negatively affect transplantation immunity. Although several humoral factors, such as C1q, TGF-β, and/or growth differentiation factor 11 (GDF11), have been considered potential candidates for promoting senescence and/or rejuvenation, androgens (e.g., testosterone, dihydrotestosterone, and dehydroepiandrosterone) are also well-known classical factors that are related to cellular senescence. Additionally, fluctuating female hormones may potentially modulate cellular senescence and SASP. Therefore, it can be concluded that the factor of biological sex should no longer be overlooked in the relationship between cellular senescence and immune-competent cells. It should be noted that this mini-review has a limitation; it does not solely describe the immune response in organ transplantation based on the relationship between antigen-presenting cells and T cell lineages, as B cells are also key players in producing antibodies to donor antigens. Further studies are needed to clarify how immune senescence affects organ transplantation in the context of biological sex difference, and these results can offer a situation-specific treatment, such as combining the treatment with senolytic agents, for transplant recipients to have favourable outcomes, according to sex and age.
